# Ureteroarterial Fistula from Ureteral Stump: A Challenging Case

**DOI:** 10.1155/2014/514625

**Published:** 2014-11-13

**Authors:** Pietro Pozzilli, Massimo Lenti, Stefano Mosca, Elisabetta Nunzi, Luigi Mearini

**Affiliations:** ^1^Diagnostic Imaging Department, Hospital S. Maria della Misericordia, University of Perugia, 06100 Perugia, Italy; ^2^Vascular and Endovascular Surgery Department, Hospital S. Maria della Misericordia, University of Perugia, 06100 Perugia, Italy; ^3^Urology Department, Hospital S. Maria della Misericordia, University of Perugia, 06100 Perugia, Italy

## Abstract

Ureteroarterial fistula (UAF) is a relatively rare condition with about 150 cases reported in the literature. Since it is a potentially life-threatening condition, a prompt diagnosis and treatment are crucial. We present here a rare, challenging case of UAF diagnosed after left nephrectomy, thus involving the ureteral stump. The difficult diagnosis and treatment by contemporary use of endovascular stent placement and ureteral occlusion by mean of metallic coils and Onyx injection are discussed.

## 1. Introduction

Ureteroarterial fistula (UAF) [1], a relatively rare condition, has been related to the use of chronic ureteral stenting for retroperitoneal fibrosis [2] secondary to pelvic external beam radiotherapy [3] and/or extensive pelvic surgery with lymphadenectomy [4, 5].

The chronic use of ureteral stent is accomplished by changes on ureteral wall induced by the foreign body [6] and periodic stent substitution progressively increases the damage at ureteroiliac passage [7]. The occurrence of gross, intermittent hematuria in the context of the clinical trial of radiation, pelvic surgery, and ureteral stenting is highly suspected as being UAF. In 2005, Krambeck et al. [8] proposed an exhaustive evaluation and treatment algorithms for the proper diagnosis and treatment of UAF.

We present here a challenging case of UAF detected after left nephrectomy and involving the ureteral stump. The proper diagnosis and treatment by mean of endovascular stent placement and endoureteral occlusion by mean of metallic coils and Onyx injection are discussed.

## 2. Case Report

A 43-year-old woman underwent open hysterectomy in 2000 for cervical squamous carcinoma, followed in 2002 by salvage lymphadenectomy and chemoradiotherapy for disease relapse. In 2007 she was diagnosed with retroperitoneal fibrosis (RF), conservatively treated with long-term placement of indwelling catheters (8-F double-J catheter) despite her young age, good prognosis, and the possibility of definitive treatment. In January 2014, the patient was admitted in another hospital for the occurrence of massive haematuria and anemia. A CT scan revealed left renal hemorrhage with cloths invading the pelvis and calyces, in absence of active vascular bleeding. She underwent open surgical exploration revealing a massive renal infarction and resulting in left nephrectomy. Despite this, gross hematuria persisted.

**Figure 1:**
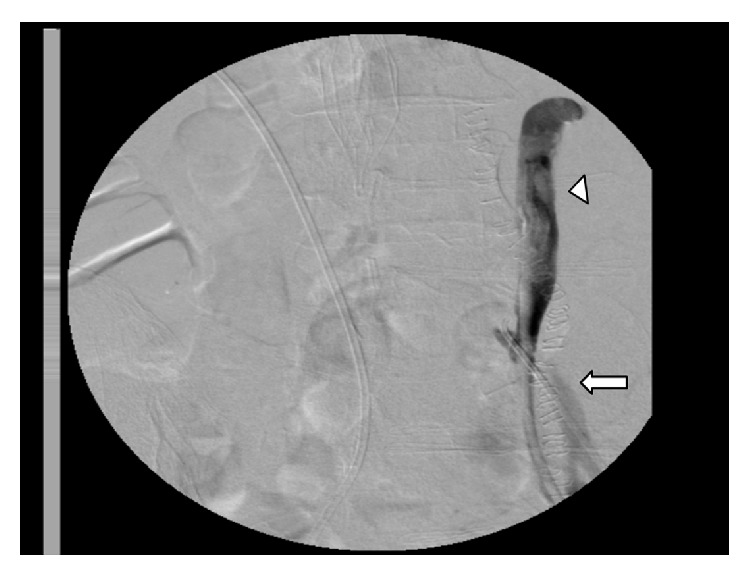
Contrast injection in left ureteral stump (arrowhead) showing extravasation in left common iliac artery (arrow).

At admission in our department, she was hemodynamically stable, although intermittent gross haematuria was present. Cystoscopy showed intermittent bleeding from left ureteral orifice; the results of CT scan and elective aortography were unremarkable. The fluky occurrence of hemodynamic stability permitted us to examine in depth the cause of ureteral bleeding, and the patient was scheduled for left ureteroscopy. During the procedure, conducted under fluoroscopic guidance, an unexpected passage of contrast medium in left common iliac artery was identified, thus revealing a UAF. The ureteral catheter was left in place, reaching the upper portion of the ureteral stump, while an intraoperative selective angiography of left common artery was repeated (Figure 1).

**Figure 2:**
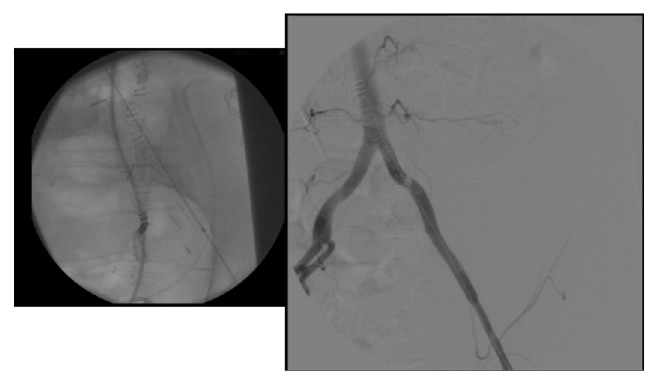
Deployment of a stent-graft on left common iliac artery.

**Figure 3:**
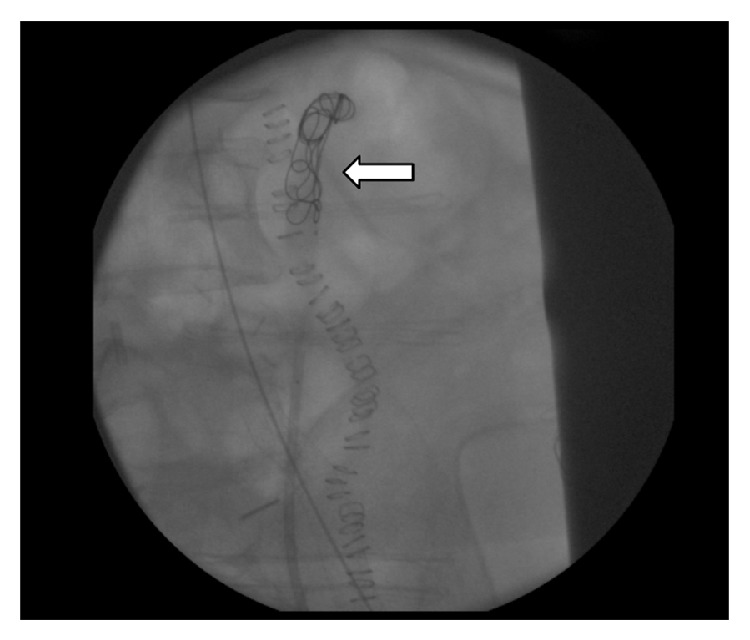
Occlusion of ureteral stump by mean of metallic coils (arrow), followed by Onyx injection.

A stent-graft measuring 10 cm in length and 9 mm in diameter (Gore Viabahn endoprosthesis with propaten bioactive surface; W. L. Gore & Associates Inc., AZ, USA) was used to treat the arterial side of the fistula on the left common iliac artery (diameter 8.5–9.2 mm on angiography, Figure 2). To prevent endoprosthesis infection the left ureteral stump was managed and closed by mean of metallic coils (Concerto Detachable Coil System; Covidien AG, Neuhausen am Rheinfall, Switzerland) followed by injection of liquid embolic system (Onyx LES; Covidien AG, Neuhausen am Rheinfall, Switzerland; Figure 3).

The procedure and hospital stay were uneventful. Hematuria disappeared suddenly after the treatment and did not recur at 8-month followup. No lower extremity complications occur.

## 3. Discussion

The occurrence of UAF has been related to the use of chronic ureteral stenting for retroperitoneal fibrosis secondary to pelvic external beam radiotherapy and/or extensive pelvic surgery. Periodic stent substitution progressively increases the damage at ureteroiliac passage, since the fibrotic changes induced by the inflammatory response to pelvic surgery or radiation therapy may fix the ureter to the artery at their crossing site. Moreover, the physical stimuli from ureteral stents with arterial pulsation may weaken the walls of both the artery and the ureter [9]. In long-surviving cancer patient, definitive surgical correction is a sustainable alternative to chronic ureteral stenting, preventing the occurrence of UAF.

Accordingly, the presentation of UAF often occurs at stent exchange and therefore UAF is highly suspected if hematuria occurs in the context of the clinical trial of radiation, pelvic surgery, and ureteral stenting, as in our patient.

The extravasation into the ureteral lumen should be certified by a complete diagnostic imaging; in emergency, patient underwent CT scan only, showing the presence of massive clots in left upper urinary tract together with kidney haemorrhage by retrograde blood flow into the kidney and collecting system. The lack of optimal scan delays showing contrast-enhancement inside the collecting system, the presence of kidney haemorrhage due to high pressure-retrograde blood flow within the collecting system, and the presence of haemodynamic instability (which probably prevented the implementation of the selective angiography) lead to surgical exploration and worthless left nephrectomy, which was followed by recurrence of haematuria. Despite the persistence of undiagnosed fistula repeated CT scan and selective angiography were again inadequate to demonstrate the presence of ureteroarterial communication at the ureteral stump level. Only the endoscopy of the ureteral stump with high-pressure contrast injection, acting as a provocative retrograde pyelography, permitted detecting an UAF. During high-pressure ureteroscopy there is the possibility of exacerbating hemorrhage; thus it has been undertaken with the support of a multidisciplinary team ready for emergent treatment.

Definitive UAF treatment involves treatment of the artery and the ureter. Arterial treatment included endovascular stenting for poor surgical candidates, arterial occlusion with or without extra-anatomic bypass for marginal candidates, and primary open arterial repair and ureteral procedure (reimplantation, psoas hitch, and Boari flap) for adequate candidates. Ureteral management includes nephrostomy tube drainage, maintenance of an indwelling ureteral stent, nephroureterectomy, or ureteral ligation with nephrostomy tube drainage in poor surgical candidates.

In our case, the vascular side of the fistula was successfully managed by endovascular stent, taking care to prevent lower limb ischemia. The ureteral side of the fistula was more difficult to manage, since no case of bleeding in the ureteral stump was reported. According to the experience of interventional radiologist, and in order to prevent fistulas' recurrence or infection of the endovascular stenting, we decided to manage the ureteral stump by the contemporary use of metallic coils followed by injection of a liquid embolic system. The combination of Onyx and coils may offer some advantages over the use of coils alone [10], since Onyx should fill the interstices between coil mass, preventing recurrence of the fistula on the ureteral side.

In conclusion, the occurrence of clinical trial of radical pelvic surgery, pelvic radiation, and chronic ureteral stenting for ureteral obstruction increases the risk of UAF, which requires a prompt diagnosis and management. The occurrence of a UAF fistula at ureteral stump, which is the new coming from current experience, is challenging in terms of diagnostic flow chart and management. The combination of endovascular stent placement and ureteral stump occlusion by means of metallic coil and Onyx permitted resolving a singular, life-threatening condition with excellent long-term results.
